# A Literature Review of *Artocarpus lacucha* Focusing on the Phytochemical Constituents and Pharmacological Properties of the Plant

**DOI:** 10.3390/molecules27206940

**Published:** 2022-10-16

**Authors:** Panal Sitorus, Jane Melita Keliat, Vivi Asfianti, Mahatir Muhammad, Denny Satria

**Affiliations:** 1 Department of Pharmaceutical Biology, Faculty of Pharmacy, Universitas Sumatera Utara, Medan 20155, Indonesia; 2 Doctoral Programme, Faculty of Pharmacy, Universitas Sumatera Utara, Medan 20155, Indonesia

**Keywords:** *A. lacucha*, phytochemicals, pharmacological activity, active compound

## Abstract

Studies have shown that approximately two-thirds of the plant species in the world have some medicinal value. *Artocarpus lakoocha* is a synonym for *Artocarpus lacucha* and is a plant that can be found in Indonesia. This medicinal plant has been used to treat many diseases. (1) Objective: This article discusses the scientific investigations carried out on *A. lacucha*, namely the plant’s chemical content, pharmacological activity, and active compounds. (2) Methods: The design of this study was based on an article that was a review of previous research. A search for relevant publications over the past ten years (2012–2022) using data from Pubmed, Proquest, Ebsco, ScienceDirect, and Google Scholar resulted in the discovery of 369 articles. (3) Results: Fifty relevant articles investigate *A. lacucha’s* substances and their applications in the health field. The presence of secondary metabolites and bioactive compounds has been reported, which is evidence that *A. lacucha* possesses antidiarrheal, immunostimulant, anticholesterol, and hepatoprotective agents. (4) Conclusions: Mobe (*A. lacucha*) is a plant native to North Sumatra, Indonesia. This plant is efficacious as an antioxidant, antibacterial, antidiarrheal, anti-inflammatory, analgesic, antinociceptive, schistosomicidal, hepatoprotective, neuroprotective, cytotoxic, antiglycation, and anticholesterol, and can also be used for anti-aging and wound healing. In addition to its various benefits, it turns out that this plant also has many active compounds that are useful to the health sector, especially the pharmaceutical field.

## 1. Introduction

In Indonesia, where there are many different ethnic groups, plants are used as medicines in increasingly different ways [1]. The number of medicinal plant species in Indonesia is unknown; hence, it is important to document their uses [2]. The use of natural substances as drugs (biopharmaceuticals) has expanded due to a back-to-nature approach adopted by many people as well as the economic crisis, which has reduced people’s purchasing capacity for pricey contemporary medicines. Natural therapies offer few negative effects. Local wisdom helps rural cultures employ plants as medicines [3].

Antidotes made from plants have been used for thousands of years. Our ancestors used them and passed them down from generation to generation until they finally made it into the pharmaceutical world and were recognized by science [4]. Many people choose to grow medicinal plants because they can be used to keep people healthy and also have other uses, for example, as nutritional supplements, cooking spices, and attractive plants [5]. Indonesia has a great opportunity to develop new drug candidates made from medicinal plants [6].

**Figure 1:**
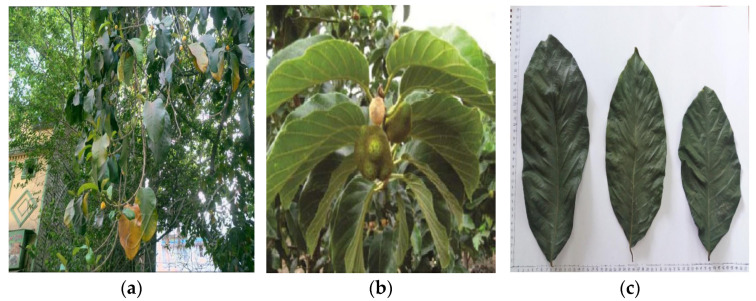
*A. lacucha* tree (**a**); *A. lacucha* fruit (**b**); *A. lacucha* leaves (**c**).

The effectiveness of medicinal plants comes from the active compounds they contain. These active compounds show up in medicinal plants that grow in places with the right climate and soil [7]. Traditional medicines have used natural ingredients for thousands of years. Several herbal extracts have been shown to lower the number of bacteria that cause cavities in the mouth [8]. *A. lacucha* is a species of *Artocarpus* from the Moraceae family (Figure 1) that contains many phenolics (flavonoids and phenolic acid). Phenolic compounds include flavonoids, phenolic acids, or phenolic derivatives [9,10,11,12]. *A. lacucha* plants have biological properties such as antiviral (for HSV and HIV), antibacterial, antimalarial, antituberculosis, antiplasmodial, antiatherosclerotic, antifungal, antidiarrheal, antidiabetic, wound healing, anti-inflammatory, and anticancer and also contain active compounds such as artocarpin, oxyresveratrol, phenols, and flavonoids [13,14,15,16,17].

## 2. Results

The articles found in this systematic review were published between 2012 and 2022. The research in the articles was carried out in a number of different countries and under laboratory conditions. The articles that met the criteria were then summed up. Based on the results of the search, 50 articles that met the criteria were found.

## 3. Methods

### 3.1. Design

A literature review is the method used in this study. A literature review tries to find gaps in the research and is conducted by analyzing and compiling what is already known about the topic being studied. A literature review is a systematic and clear way to evaluate and synthesize the research and critical thinking results produced by researchers and practitioner reviews [18].

### 3.2. Article Criteria

In order to narrow down the search for the review papers, criteria for inclusion were established in this study. The inclusion criteria were (1) a focus on mobe (*A. lacucha*), (2) related to the subject of medicine, (3) full texts, (4) articles published between 2012 and 2022, (5) original research articles, and (6) English language articles. The exclusion criteria were (1) articles that only contained an abstract, (2) incomplete texts, and (3) double publications. The question of this study was how often scientifically sound testing of *A. lacucha’s* biological activity, active compounds, and pharmacological effects is conducted.

**Figure 2:**
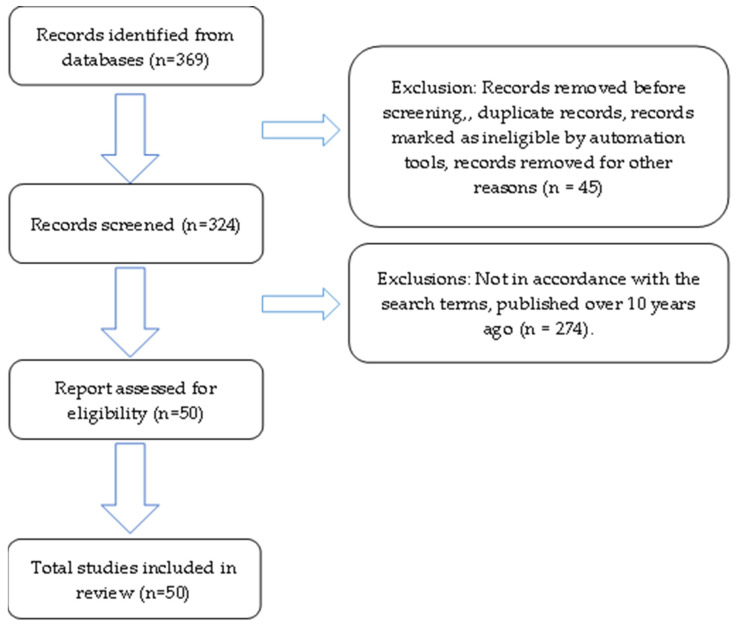
Flow diagram demonstrating the screening method for the article.

A total of 369 articles were found using search engines including PubMed, Ebsco, ProQuest, ScienceDirect, and Google Scholar. Based on the search engine results, 50 articles were found that met the inclusion criteria; 22 articles on the biological activity of A. lacucha, 9 articles on the active compounds contained in A. lacucha, 7 articles on the nutritional content of A. lacucha, and 12 articles on the pharmacological activity of A. lacucha. The items that satisfied the criteria were then compressed. The conclusions of the summary are thoroughly discussed later in this paper. The selection process can be seen in Figure 2.

### 3.3. Article Search

In the search, combinations of specific words, such as “Mobe”, “*Artocarpus lacucha*”, “Pharmacological”, “Compound”, and “Activity”, were used. This search used search engines such as PubMed, Ebsco, Proquest, ScienceDirect, and Google Scholar.

### 3.4. Study Selection

As shown in Figure 2, the prism technique was used to select the articles, beginning with the identification, screening, and eligibility and concluding with the included items.

## 4. Discussion

The mobe plant, which is also called *A. lacucha*, has a lot of good qualities. The active compounds that can be taken from it can be used in the pharmaceutical industry as raw materials to make new drugs. So, the mobe plant is a species that is thought to be very important because of its many benefits [19].

### 4.1. Nutrient Content in A. lacucha

Adequate plant nutrition is vital during the overall growth period. Plant nutrition is related to how plants obtain, distribute, and use the nutrients in the soil. It is also related to the processes and reactions in a plant body used for its growth and development. Plants generally obtain nutrients from the soil solution [20,21]. The nutrients are obtained in the form of primary nutrients, secondary nutrients, and micronutrients. Nutrients are converted into cellular material or used as an energy source in what is known as the metabolic processes. Metabolism produces the metabolites needed for plant growth and development [22]. The term metabolism includes the various reactions in living cells for maintaining life and growth. Thus, nutrients and metabolism are reciprocal [23]. Plants need complete nutrients to grow and develop correctly and produce quality products. Nutrients are the primary needs of plants that support their growth. If the soil elements are unavailable, plant growth will be hampered and production will decrease. Plants’ requirements for nutrients already exist in the soil but their availability is sometimes insufficient to meet plants’ needs [19,24].

The *A. lacucha* fruit is a medicinal fruit that can be consumed as part of a staple diet. According to reports, fresh equivalents are high in water (82%) and fiber (2%) and contain a significant amount of vitamin A (423 IU) and vitamin C (135 mg/100 g) (Table 1). Monkey fruit is important in the human diet due to its high concentrations of vitamin C and carotene [25,26]. According to one source, *A. lacucha* fruit contains a significant number of macro- and microminerals such as calcium (66.6 mg), magnesium (23.6 mg), potassium (350 mg), phosphorus (22.1 mg), iron (778 μg), zinc (3981 μg), copper (7974 μg), and manganese (2025 μg) [27,28]. They bind with C, H, O, and S to form amino acids, the protein building blocks. Amino acids generate protoplasm, which plants need for cell division and growth. Protein-based plant enzymes require nitrogen (N) for all enzymatic processes. Photosynthesis requires N because it is part of the chlorophyll molecule. Vitamins need N and N boosts green vegetable dry matter and grain protein [29]. These elements can come from organic or inorganic sources. Nutrients are inorganic parts of the soil that plants need to grow and develop. Elements such as sulfur (S), calcium (Ca), phosphorus (P), iron (Fe), molybdenum (Mo), boron (B), manganese (Mn), zinc (Zn), oxygen (O), and carbon (C) are part of the essential elements group. Non-essential elements, on the other hand, do not play a big role in plant growth so other elements can take their place. Sodium (Na), silicon (Si), bromine (Br), and flour make up this group of non-essential elements [30].

Herbaceous plants are 90% water and 10% carbon, hydrogen, and oxygen. A minor part of dry matter, but an important fraction, consists of 13 necessary nutrients for higher plants. The 13 essential nutrients are separated into two groups based on the amount plants need and are the macronutrients and micronutrients (N, P, K, Ca, Mg, and S). Micronutrients are needed in small levels, indicated as ppm per unit of dry matter (Fe, Mn, Zn, B, Mo, Co, and Cl). Chemically examining healthy plants can help establish which nutrients are required and how much of each is needed [31].

### 4.2. Antioxidant Activity of A. lacucha

Antioxidants counteract the free radicals created by the body’s chemical reactions and metabolic processes. Free radicals are highly reactive intermediate chemical entities with an unpaired electron [32]. Free radicals can live on their own for short amounts of time. In the body and food systems, different oxygen-free radicals and other reactive species can form [33].

Antioxidants stop or break the chain reaction of free radicals in the body so that they do not harm the body’s cells. Antioxidants can be made in a lab or they can be found in nature. Synthetic antioxidants work well but they are not always safe [34]. Natural antioxidants are safe to grow because they are not contaminated with chemicals [35]. Animal studies have shown that phytochemical antioxidants in the diet can help to eliminate free radicals. Phenolics are a large group of different secondary plant chemicals that are found throughout the plant kingdom. Compounds with several or many phenolic hydroxyl substituents are often referred to as polyphenols [32].

Based on the results of the literature study, the antioxidant activity of various solvents and antioxidant methods, as shown in the table below about *A. lacucha* leaf extract with methanol and ethanol as solvents using the DPPH, ABTS, FRAP Hydroxyl, and Superoxide anion methods; an IC_50_ value ≤ 50 indicates that it has strong activity [36,37,38,39,40], whereas the methanol fraction from *A. lacucha* seeds using the DPPH, ABTS, and FRAP methods obtained IC_50_ values of 100–150 in the medium category and IC_50_ ≥ 150 in the weak category in trapping free radicals [39,41]. To test the presence of antioxidant activity, one can use the DPPH method. The observation of DPPH radical scavenging can be carried out by observing the decrease in absorbance. This can occur due to radical reduction by antioxidants (AH) or reaction with other radical compounds [42]. Free radical scavengers are the primary mechanism by which antioxidants react in food. One way to test the activity of a compound as an antioxidant is to react it with the DPPH reagent spectrophotometrically [43]. The DPPH method is not specific to a particular antioxidant component but to all antioxidant compounds in the sample. The measurement of the total antioxidant capacity can help understand the functional properties of food. The DPPH method was chosen because it is simple, easy, fast, sensitive, and only needs a small sample (Table 2).

Potassium persulfate acts as an oxidizing agent and reacts directly with ABTS to produce ABTS^+^ in high yields. Then, antioxidants react with ABTS^+^, which makes the color of the ABTS^+^ radicals lighter. With potassium persulfate, the reaction that produces free radicals from ABTS becomes ABTS^+^, and the reaction that eliminates the free radicals with the antioxidants makes ABTS stable again [44] (Table 2).

The FRAP (Ferric-Reducing Antioxidant Power) method is a simple and fast way to assess antioxidant activity. To figure out how many antioxidants there are, simple reagents are used and no special tools are needed. The main idea behind this method is that antioxidant compounds change ferric ions into ferrous ions. The FRAP method is based on a compound’s ability to change potassium ferricyanide (K_3_Fe(CN)_6_) into potassium ferrocyanide (K_4_Fe(CN)_6_). By giving up an electron, the antioxidants in the sample will turn Fe^3+^ into Fe^2+^. If the sample is measured at a wavelength of 700 nm, it is possible to find out how many Fe^2+^ complexes are in it [43]. In biological systems, hydroxyl radicals can be made when hydrogen peroxide (H_2_O_2_) reacts with iron ions (Fe^2+^). This is called the Fenton reaction. 

The total level of phenols and flavonoids causes the differences in antioxidant activity that have been found. The antioxidant activity of phenolic and flavonoid compounds is linear so the more of them there are, the better they are [45,46]. Because of this, it is thought that the high total phenolic content of the extract plays an important role as an antioxidant. Tannins and other phenolic compounds such as flavonoids are also known to have antioxidant properties [47]. Alkaloids and terpenoids, which are other types of secondary metabolites, also have antioxidant properties. The flavonoids, tannins, alkaloids, terpenoids, and organic sulfur compounds in phenolic compounds, alkaloids, and terpenoids all act as natural antioxidants. However, antioxidant activity is not always linked to the number of phenols or flavonoids. This can happen for a number of reasons such as differences in the active parts of plants, synergistic or opposing effects between the active parts, research conditions, and methods used, all of which can affect antioxidant activity in plants [48].

### 4.3. Antimicrobial Activity of A. lacucha

Antimicrobials are materials or drugs used to eradicate microbial infections in humans. Medications used to eliminate microorganisms that cause disease in humans, animals, or plants must be selectively toxic, meaning that the drug or substance must be highly toxic to disease-causing microorganisms but relatively non-toxic to the host’s body. An ideal antimicrobial agent has selective toxicity. This means that a drug harms the parasite but does not harm the host. Often, selective toxicity is relative rather than absolute; this means that a prescription at a certain concentration can be tolerated by the host and can damage the parasite. An ideal antibiotic as a drug must meet certain requirements [49].

Based on the literature study, Table 3 shows the antimicrobial activity of the aqueous extracts of the leaves, bark, and wood of A. lacucha that can inhibit the growth of bacteria and fungi with an average inhibition zone of 10–20 mm in the strong category and ≥20 mm in the very strong category. Meanwhile, A. lacucha wood ethanol extract also inhibited the bacterial inhibition zone in the strong category with an average of 10–20 mm [16,50,51]. The determination of the sensitivity of pathogenic bacteria to specific antibacterial agents can be conducted using one of two primary methods, the dilution method or the diffusion method. There are several methods used for measuring bacteria, but the most commonly used are the dilution and diffusion methods. These methods measure the minimum inhibitory concentration (MIC) and minimum kill rate. For these methods, a series of antimicrobial dilutions are made on media to which the test microbes have been added. The test solution used for antimicrobials is called the MIC at the lowest level and looks clear, with no signs of microbial growth [52]. The MIC solution is re-cultured for 18–24 h without testing microorganisms or antimicrobial agents. Most antimicrobial tests involve agar diffusion. A medication is placed on an inoculated solid medium and incubated for 18–24 h. The buffer’s zone of inhibition diameter is used to measure the drug’s microorganism resistance. This approach is affected by its medium nature, diffusion capacity, molecular size, and drug stability. Standardization allows for a good sensitivity test [53,54].

After 1 × 24 h of incubation, observations are made. The diameter of the inhibition zone shows how sensitive bacteria are to antibiotics or other antibacterial materials used as a test material. This is shown by the size of the clear area. First, a scale ruler is used to measure the diameter of the inhibition zone in millimeters (mm). Then, based on the Davis and Stout classification, the size of the inhibition zone is used to figure out how well it killed bacteria [55]. The following are used to measure antibacterial power: a weak inhibition zone has a diameter of 5 mm or less, a moderate inhibition zone has a diameter of 5–10 mm, a strong inhibition zone has a diameter of 10–20 mm, and a very strong inhibition zone has a diameter of 20 mm or more [56].

Tannins and flavonoids are two types of chemical compounds that can make something antibacterial. Phenolic compounds include tannins and flavonoids. It is known that the phenol group kills bacteria but this does not happen randomly. Even though phenolic compounds kill bacteria by changing the shape of cell proteins and damaging bacterial cell walls, they can also precipitate proteins and damage lipids in cell membranes by lowering the surface tension of cell membranes. Flavonoids kill bacteria by hurting their cytoplasmic membranes. The cytoplasmic membrane of a bacterium controls how much food or nutrition can enter [57]. Flavonoids can also combine with proteins outside of cells to make complex molecules. These molecules can cause proteins to clump together, which can stop bacterial cells from growing. Tannins, on the other hand, can damage and shrink the bacterial cell wall, which makes the cell less permeable. Because of this, cells cannot do anything to stay alive and their growth slows or stops [58].

### 4.4. Pharmacological Activity of A. lacucha 

The cytotoxicity test is an in vitro toxicity test using cell culture and is used in evaluating the safety of drugs, cosmetics, food additives, and pesticides and to detect the presence of the antineoplastic activity of a compound. Cytotoxic compounds are toxic to tumor cells in vitro and can also be toxic to normal cells that proliferate rapidly. These compounds have antitumor activity if this toxicity is transferred across tumor cells in vivo. *A. lacucha* plants have various biological activities, one of which is cytotoxic. Based on the information collected, a comparison of the effects of the different concentrations of the ethanol extract of *A. lacucha* leaves of 10, 20, 40, 60, 80, and 160 μg/mL [59] and the pericarp methanol extract with a concentration of 10–1000 μg/mL [25] obtained an LC_50_ of antioxidant activity, which was determined by regression analysis. *A. lacucha* extract was found to kill brine shrimp in a way that changed with the amount given. There may be saponins, alkaloids, and cardiac glycosides in the extract, which could explain why the brine shrimp were killed by it [25].

*A. lacucha* leaves extracted in methanol were found to have anti-inflammatory effects that were dose-dependent and statistically significant (*p* ≤ 0.05). A. lacucha was more effective at stopping inflammation than indomethacin at a dose of 200 mg/kg (64.90%). The writhing response was stopped by 29.63% and 57.41%, respectively, by the dose of *A. lacucha* leaf methanol extract (*p* ≤ 0.05) (Table 4). One thing that can cause inflammation is the byproduct of the breaking down of arachidonic acid. Arachidonic acid is a type of fatty acid that has 20 carbon atoms and is not saturated. Physical, chemical, and biological stimuli turn on cell phospholipases, which release arachidonic acid from phospholipids [40].

Analgesics, also called pain blockers, are drugs that stop or lessen pain without a loss of consciousness. At doses of 50–200 mg/Kg b.w., hydro-methanolic *A. lacucha* wood extract has been shown to have a strong effect against pain (Table 4), and was not harmful to the animals used in the experiments. Nociceptive pain is a type of pain that disappears as soon as the affected part of the body heals. Opioid painkillers work well for treating both acute and chronic pain [60]. Opioid analgesics reduce pain but people can develop an addiction, respiratory depression, hypotension, tolerance, and dependency [61]. So, ingredients from nature that have antinociceptive activity are used as an alternative to opioid painkillers to lessen the side effects of pain.

The leaves of the *A. lacucha* plant (100 mg/kg) show that it has a moderate effect as an antidiarrheal. This is based on the data that were collected. At a dose of 200 mg/kg, on the other hand, it was stopped (68.11%), which is close to what the standard drug loperamide can do (71.1%). So, the given extract stopped diarrhea through an antisecretory mechanism. This was also shown by the fact that the number of wet stools in the experimental test group decreased. The goals of therapies for diarrhea are to fix the diet, stop losing too much water and electrolytes, fix acid-base problems, treat the symptoms, treat the causes of the diarrhea, and treat the diseases that cause diarrhea as a side effect. So, diet management is the most important aspect of treating diarrhea [62].

*A. lacucha* plants also have other pharmacological effects such as protecting nerve cells and the liver. The neuroprotective effect of the aqueous extract of *A. lacucha* wood suggests that mitochondrial protection is hard to find, even though the two drugs studied, puaghaad and oxyresveratrol, improve cell survival, especially ROS levels and lipid peroxidation. The effect of oxyresveratrol on the expression of redox-sensitive antioxidant enzymes and their pharmacokinetics suggests that oral puaghaad may be a great way to protect against transient neurodegenerative diseases (Table 4) [63]. Quantitatively, several studies have determined oxyresveratrol levels using the TLC densitometric method and the qNMR method [64,65]. Mice given paracetamol were given hepatoprotection in real life, and silymarin was used as a control at 100 mg/kg/day for eight days. In addition, mice were given 125, 250, and 500 mg/kg/day of *A. lacucha* methanol extract. At 125 and 250 mg/kg/day, the mice that were given the extract showed mild swelling, steatohepatitis, moderate neutrophil and leukocyte infiltration, and geographic necrosis. At the same time, steatohepatitis and necrosis were not seen at a dose of 500 mg/kg [66]. Neuroscientists have become very interested in the idea of neuroprotectors in the last few decades, as neurological disorders that affect the brain, spinal cord, and nerves that connect them have become more common. At first, free radicals were thought to be the cause of this disorder. However, different pathomechanisms were later linked to different diseases [67].

**Table 4:** Pharmacological activity of *A. lacucha* based on scientific data.

Sample	Activity	Method	Result	Reference
*A. lacucha* leaf methanol extract	Cytotoxic	Brine shrimp lethality bioassay	The extract has a strong toxic potential. LC_50_ value of the extract was 2.83 ± 0.11 μg/mL.	[59]
	Anti-inflammatory	Carrageenan-induced paw edema test in mice	The extract exhibited anti-inflammatory effects at a 200 mg/kg dose.	
	Analgesic	Acetic acid-induced writhing test	The extract inhibited 29.63% and 57.41%.	
	Antidiarrhoeal	Castor oil-induced diarrhea	The extract decreased castor oil-induced diarrhea of the test animals at both 100 and 200 mg/kg doses.	
*A. lacucha* fruit methanol extract	Hepatoprotective	In vivo-induced paracetamol in mice	The extract prevented increases in liver function tests and paracetamol-related histopathological alterations.	[68]
*A. lacucha* heart wood aqueous extract	Neuroprotective	H_2_O_2_-induced oxidative stress in SH-SY5Y cells	The extract has neuroprotective activity.	[63]
*A. lacucha* heart pericarp methanol extract	Cytotoxic	Brine shrimp lethality bioassay	The extract was found to be toxic with an LC_50_ of 427.74 μg/mL.	[25]
*A. lacucha* crude aqueous extract	Schistosomicidal	In vivo mice infected with *Schistosoma* *mansoni*	The extract at a concentration of 250 μg/mL exhibited reduced motility.	[54]
*A. lacucha* bark hydro-methanolic extract	Antinociceptive	Tail immersion, hot plate, acetic acid, formalin-induced nociception, and carrageenan-induced paw edema tests	The extract has antinociceptive activity.	[11]
*A. lacucha* leaf methanol extract	Anticholesterol	In vivo, hyperlipidemia-induced rats were given an extract	The extract significantly lowered the serum total cholesterol, triglycerides, and low-density lipoprotein (LDL) levels, while effectively increasing serum high-density lipoprotein (HDL) levels.	[69]
*A. lacucha* heart wood aqueous extract	Antiglycation	Bovine Serum Albumin (BSA)	The extract inhibited AGE-BSA.	[29]
*A. lacucha* leaf ethanol extract	Proliferative and Wound healing	MTT assay and in vivo, NIH-3T3 cells in mice	The extract had a proliferative effect and wound-healing properties.	[44]

*A. lacucha* plants are also good for lowering cholesterol, killing schistosomes, reducing pain, preventing diabetes, and healing wounds. The anticholesterol activity was tested on rats in the wild by giving them 250 mg/kg of a methanol extract and 10 mg/kg of simvastatin as a control. Compared to the diseased group, the results showed that total serum cholesterol, triglycerides, and low-density lipoprotein (LDL) levels decreased, whereas serum high-density lipoprotein (HDL) levels increased. Hyperlipidemia, also called hypercholesterolemia, is a condition in which the amount of fat in the blood is too high. Cholesterol diseases are becoming more prevalent because more people are eating foods with a lot of saturated fat, which is often found in fast food. Aside from food, a lack of exercise and high levels of depression can also cause increases in blood cholesterol. Mice were tested for schistosomicidal activity in vivo, and adult worms were grown in a medium with 250, 500, and 750 mg/mL. The results showed that Schistosoma mansoni could be affected by *A.* lacucha water extract with a concentration of 250 mg/mL. Schistosomiasis is one of the most important parasitic diseases in terms of public health. Many developing countries lose money and have health problems because of this disease. Schistosomiasis is caused by the worm *Schistosoma japonicum* and is found in many Asian countries [70,71]. Several other studies have shown that *A. lacucha* kills the larvae of Anopheles stephensi, Aedes aegypti, Culex quinquefasciatus, Paramphistomum cervi, and Plasmodium falciparum. The larvacidal bioassay was used to conduct this test [54,72,73].

At 1 mg/mL, the antiglycation activity of *A. lacucha* wood water extract inhibited AGE-BSA formation by 46.3% (96 h), 67.8% (168 h), and 71.3% (192 h). At 10 mg/mL, it inhibited AGE-BSA formation by 100% (96 h), 95.5% (168 h), and 100% (192 h). At 100 mg/mL, *A lakoocha* extract also inhibited the formation of AGE-BSA (192 h) (Table 4) [74]. Advanced glycation end products (AGEs), which cause aging, are produced when reducing sugars react with amino groups in proteins, nucleic acids, or phospholipids without the help of enzymes [75]. The ethanol extract of *A. lacucha* leaves at a concentration of 37.5 μg/mL sped up the process of wound healing in NIH-3T3 cell lines by making more cells divide, move, and express VEGFR-2 genes. Healing a wound is a continuous process that can be broken down into several phases. Some of these phases are blood clotting, inflammation, growth, change, and bone formation.

*A. lacucha* plants contain several active compounds, which have been discussed in this article. One of them is oxyresveratrol, which has anti-aging activity associated with its high phenol content, free radical scavenging activity, and antiglycation activity [76]). Oxyresveratrol compounds could work as antioxidants and stop the production of melanin in B16 melanoma cells. Based on what we know so far, they can also stop glucose transporters in human intestinal Caco-2 cells [77,78,79].

### 4.5. A. lacucha Active Compound

Based on the results of the literature study, it can be stated that the *A. lacucha* fruit has antibacterial, antihelmintic, and cholesterol-lowering properties (Table 5). People believe that it helps the liver, blood, and digestive system [80]. In general, this fruit has a unique sweet-and-sour taste and is a lot of fun to eat. *A. lacucha* fruit is a healthy food that can be eaten fresh when it is ripe. It is often used to make curries, pickles, chutneys, sauces, and drugs [10,13]. The risk of developing non-communicable diseases such as heart disease, diabetes, cancer, and neurodegenerative diseases may decrease if tropical fruits are consumed regularly [81]. They also contain a lot of phytochemicals and pharmacological components [82]. Other information has shown that the methanol extract of *A. lacucha* root contains 5,7,2′,4′-tetrahydroxy-6-geranyl-3-prenyl-flavone compounds, as well as three known flavonoids: afzelechin-3-O—L-rhamnopyranoside, catechin, and cudraflavone C. Using the inactivation method, these isolates were tested for their ability to stop herpes simplex virus types 1 and 2. The compound 2-Arylbenzofurans is a good source of the acetylcholinesterase (AChE) agent in Alzheimer’s disease [82,83,84].

## Figures and Tables

**Table 1:** The nutritional content of *A. lacucha*.

Constituents	Levels
Water (%)	82.1
Carbohydrate (%)	13.1
Protein (%)	0.7
Lipid (%)	1.1
Fibre (%)	2.0
Vitamin A (IU)	423
Thiamin (μg %)	0.02
Riboflavin (μg %)	0.15
Niacin (μg %)	0.3
Ascorbic acid (mg/100 g)	135
Potassium (mg/100 g)	13.50
Magnesium (mg/100 g)	23.60
Phosphorus (mg/100 g)	22.10
Calcium (mg/100 g)	66.60
Iron (mg/100 g)	0.77
Zinc (mg/100 g)	3.98
Manganese (mg/100 g)	2.02
Copper (mg/100 g)	7.97

**Table 2:** Antioxidants of *A. lacucha*.

Sample	Method	Result	Reference
*A. lacucha* leaf ethanol extract	DPPH	IC_50_ value 48.23 ± 0.46 μg/mL	[36]
*A. lacucha* leaf methanol extract	DPPH	IC_50_ value 6.72 ± 4.70 mg Ascorbic acid/gm	[37]
	ABTS	IC_50_ value 2.32 ± 1.27 mg Ascorbic acid/gm	
	Hydroxyl	IC_50_ value 41.35 ± 11.75 mg BHT/gm	
	Superoxide anion	IC_50_ value 0.47 ± 0.13 mg ascorbic acid/gm	
*A. lacucha* seed methanol extract	DPPH	IC_50_ value 18.28 ± 4.22 μg/mL	[38]
	FRAP	IC_50_ value 31.22 ± 0.89 μg/mL	
*A. lacucha* seed methanol fraction	DPPH	IC_50_ value 111.98 ± 34.20 μg/mL	[39]
	ABTS	IC_50_ value 138.26 ± 0.66 μg/mL	
	FRAP	IC_50_ value 316.81 ± 2.96 μg/mL	
*A. lacucha* leaf methanol extract	DPPH	IC_50_ value 26.95 ± 0.009 μg/mL	[40]
*A. lacucha* leaf n-hexane, ethyl acetate, and ethanol extract	DPPH	IC_50_ value n-hexane (1062.03 ± 1.42 μg/mL); ethyl acetate (323.18 ± 0.02 μg/mL); ethanol (99.23 ± 0.07 μg/mL)	[41]

**Table 3:** Antimicrobial activity of *A. lacucha*.

Sample	Bacteria/Fungi	Method	Inhibition Zone (mm)	Reference
*A. lacucha* leaf aqueous extract	*Streptococcus mutans*	MIC/MBC	30.5 ± 0.00	[16]
	*Streptococcus sobrinus*		16.0 ± 0.35	
	*Enterococcus faecalis*		18.2 ± 0.00	
	*Lactobacillus fermentum*		17.7 ± 0.35	
	*Lactobacillus salivarius*		15.0 ± 1.41	
	*Aggregatibacter actinomycetemcomitans*		29.5 ± 2.12	
	*Porphyromonas gingivalis*		21.0 ± 1.41	
	*Prevotella intermedia*		30.5 ± 0.70	
	*Prevotella nigrescens*		25.5 ± 0.70	
	*Fusobacterium nucleatum*		30.7 ± 0.35	
	*Tanerella forsythia*		29.0 ± 0.00	
*A. lacucha* bark aqueous extract	*Candida albicans* ATCC 90028	MIC/MBC	20 ± 0.13	[50]
	*Candida albicans* ATCC 10231		15 ± 0.14	
	*Candida dubliniensis* MYA-577,		15 ± 0.21	
	*Candida dubliniensis* MYA-646,		20 ± 0.15	
	*Candida glabrata* ATCC 66032		20 ± 0.13	
	*Candida glabrata* ATCC 90030		18 ± 0.06	
	*Candida krusei* ATCC 34135		13 ± 0.06	
	*Candida krusei* ATCC 6258		14 ± 0.10	
	*Candida tropicalis* ATCC 66029		28 ± 0.05	
	*Candida tropicalis* ATCC 750		19 ± 0.15	
	*Candida tropicalis* ATCC 13803		20 ± 0.06	
*A. lacucha* heartwood ethanol extract	*Corynebacterium* sp.	MIC/MBC	20.3 ± 0.6	[51]
	*Staphylococcus aureus*		16 ± 0	
	*Staphylococcus epidermidis*		20.7 ± 0.6	
	*Bacillus* sp.		16 ± 0	
	*Micrococcus luteus*		23.3 ± 0.6	
	*Pseudomonas aeruginosa*		11.7 ± 0.6	
	*Methicillin-Resistant Staphylococcus aureus*		16.7 ± 0.6	
	*Propionibacterium acnes*		15.7 ± 0.6	
*A. lacucha* wood aqueous extract	*Corynebacterium* sp.		24.0 ± 0	
	*Staphylococcus aureus*		15.7 ± 0.6	
	*Staphylococcus epidermidis*		18.3 ± 0.6	
	*Bacillus* sp.		15.7 ± 0.6	
	*Micrococcus luteus*		21.7 ± 0.6	
	*Pseudomonas aeruginosa*		9.7 ± 0.6	
	*Methicillin-Resistant Staphylococcus aureus*		17.3 ± 0.6	
	*Propionibacterium acne*		12.7 ± 0.6	

**Table 5:** Active compounds of *A. lacucha* based on scientific data.

Active Compound	Activity	Reference
Cycloartenone (1)	Antihyperglycemic, hypolipidemic, and antiatherosclerotic	[85]
α-amyrin acetate (2)	Anti-inflammatory activity; helps to decrease mechanical sensitization, hypersensitivity, and edema; radical scavenging and antihyperlipidemic activity	[86]
β-amyrin acetate (3)	Anti-inflammatory activity; helps to decrease mechanical sensitization, hypersensitivity, and edema; radical scavenging and antihyperlipidemic activity	[86]
Lupeol acetate (4)	Antioxidant activity; decreases cholesterol, phospholipid, and triglyceride levels; and interrupts cardiovascular disease	[87]
Oxyresveratrol (5)	Significantly delays the development of skin lesions and is antiviral, cytotoxic, anti-HSV, anti-HIV	[88]
Lakoochin B (6)	Anti-mycobacterial activity, cytotoxic against nasopharyngeal carcinoma and breast cancer	[89]
Lakoochin A (7)	Anti-mycobacterial activity, cytotoxic against breast cancer	[89]
Norartocarpin (8)	Antioxidant and antityrosinase activity, skin-whitening agent	[90]
Artocarpin (9)	Cytotoxic in lung cancer cells	[91]
Cycloartolakoochol (10)	Moderate activity against herpes simplex virus (HSV-1 and 2)	[92]
4-hydroxyartolakoochol (11)	Both compounds have inhibitory activity against acetylcholinesterase and butyrylcholinesterase	[92]
Cycloartocarpin (12)	Significant antiplasmodial and antitubercular properties and relative cytotoxic activity for breast cancer and human oral epidermoid cancer	[93]
Cudraflavone C (13)	Significant antiplasmodial and antitubercular activities and relative cytotoxic activity for breast cancer and human oral epidermoid cancer	[93]
Diethyl phthalate (14)	Antioxidant activity	[39]
3,4-Dihydroxymandelic acid (15)	Antioxidant and antimicrobial activity	[39]
7,8-Didehydro-3-methoxy-17-methyl-6-methylene, morphinan (16)	Antioxidant and antimicrobial activity	[39]
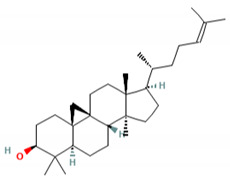	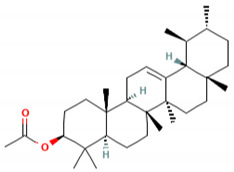	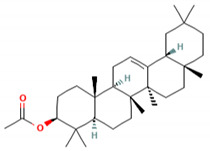
C_30_H_50_O MW = 426.7 (1)	C_32_H_52_O_2_ MW = 468.8 (2)	C_32_H_52_O_2_ MW = 468.8 (3)
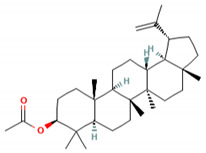	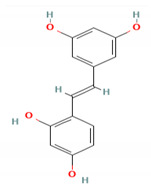	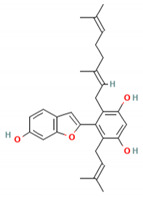
C_32_H_52_O_2_ MW = 468.8 (4)	C_14_H_12_O_4_ MW = 244.24 (5)	C_29_H_34_O_4_ MW = 446.6 (6)
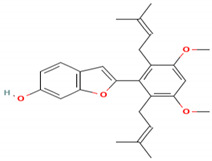	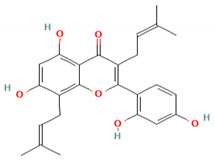	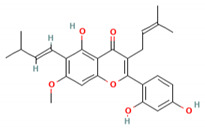
C_26_H_30_O_4_ MW = 406.5 (7)	C_25_H_26_O_6_ MW = 422.5 (8)	C_26_H_28_O_6_ MW = 436.5 (9)
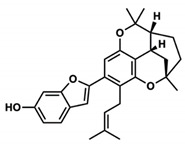	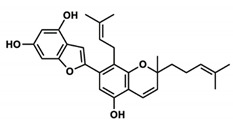	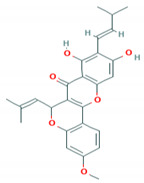
C_29_H_32_O_4_ MW = 444.23 (10)	C_29_H_32_O_5_ MW = 460.22 (11)	C_26_H_26_O_6_ MW = 434.5 (12)
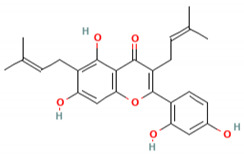	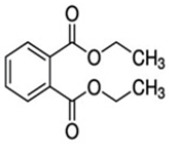	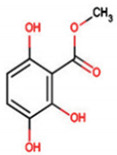
C_25_H_26_O_6_ MW = 422.5 (13)	C_12_H_14_O_4_ MW = 222.24 (14)	C_8_H_8_O_5_ MW = 184.15 (15)
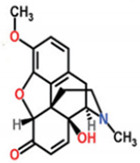		
C_19_H_23_NO MW = 281.4 (16)		
